# The size of the population potentially in need of palliative care in Germany - an estimation based on death registration data

**DOI:** 10.1186/s12904-016-0099-2

**Published:** 2016-03-08

**Authors:** Nadine Scholten, Anna Lena Günther, Holger Pfaff, Ute Karbach

**Affiliations:** IMVR – Institute for Medical Sociology, Health Services Research and Rehabilitation Science, University of Cologne, Cologne, Germany

**Keywords:** Palliative care, Palliative need, Population-based estimate, End-of-life care, Death registration data

## Abstract

**Background:**

No data exist on the size of the population potentially in need of palliative care in Germany. The aim of this study is to estimate the size of the German population that may benefit from palliative care.

**Method:**

Based on existing population-based methods (Rosenwax and Murtagh), German death registration data were analyzed and contrasted with international results. The data include all death cases in 2013 in Germany.

**Results:**

According to the method Rosenwax defined, between 40.7 % (minimal estimate) and 96.1 % (maximal estimate) of death cases could benefit from palliative care. The estimation, based on Murtagh’s refined method, results in 78.0 % of death cases potentially being eligible for palliative care. The percentage of potential palliative care candidates is conditioned by age. Based on the Murtagh Method, in the age category between 30 and 39 years, a potential demand for palliative care can be found for 40.4 % percent of all deaths occurring in this age category, with this number increasing to 80.3 % in the age bracket of 80 years and over.

**Conclusion:**

An estimation of the size of the population in need is essential for healthcare planning. Therefore, our data serve as a guide and starting point for further research.

## Background

In an aging society, the burden of dealing with cancer and other chronic life-threatening diseases increases. According to the WHO (World Health Organization), the aim of palliative care, as an interdisciplinary profession, is to provide symptom control and support for affected patients and their families who are facing a terminal illness [1]. Palliative care has been established to focus on relieving pain in cancer patients [2]. Until now, palliative care has been centered around the treatment of cancer patients [3, 4], although there has been increasing research showing the demand for palliative care in chronic non-cancer patients with a terminal diagnosis [5–7]. The need for palliative care can be defined in many ways [8], including using Bradshaw’s categories (felt, expressed, normative, and comparative needs) [9]. The ability of patients to benefit from palliative care (normative perspective) seems to be a useful approach for defining need in this context [10]. In Germany, the majority of patients in palliative care services are cancer patients [3]. Because terminally ill patients with cancer, as well as those with non-cancer diagnoses, benefit from palliative care, it is important to enable access for all patients that could benefit from palliative care [5–7]. In order to provide the needed care and to make adequate palliative care services available, it is necessary to have data on the actual need for care. Regarding palliative care, there is a distinction to be made between primary palliative care provided by general practitioners, for example, or specialized palliative care supplied by physicians specializing in palliative care, for example in hospitals or ambulatory teams [11].

## Palliative care in Germany

Palliative care is an emerging medical field in Germany, but compared to Great Britain, which is considered the pioneer in the development of palliative care [12], more action needs to be taken to catch up, even though Germany is improving [13]. According to the Quality of Death Index 2015, published by the *Economist*, Germany ranks number 7 behind Great Britain (No. 1), Australia (No. 2) and New Zealand (No. 3) [14]. Palliative care has been stated to be a human right [15]. With the healthcare reforms in 2007 in Germany, every patient in need of specialized palliative care should be able to receive that care [16].

## Population based methods to estimate need for palliative care

Based on Western Australian death record data, Rosenwax et al. [17] developed a method in 2005 to estimate the minimal, mid-range, and maximal numbers of people that could potentially benefit from palliative care during their last year of life based on administrative data. For the minimal estimate, 10 medical conditions (neoplasms, heart failure, renal failure, chronic obstructive pulmonary disease, Alzheimer’s disease, liver failure, Parkinson’s disease, motor neuron disease, Huntington’s disease, and HIV/AIDS) were determined using focus groups, interviews, and literature reviews, identifying affected patients who could benefit from palliative care before death. The codes for these medical conditions are used to identify the population of individuals that are potentially in need for palliative care within death registration data. In other words, everybody who died because of one of the defined conditions is classified as a palliative care candidate. The mid-range estimate resulted from matching death certificate data with hospital data, including certain hospital admissions during the last year of life. The maximal estimate is based on all deaths, excluding death during pregnancy, childbirth, or the puerperium, originating during the perinatal period, or resulting from external causes, like injury or poisoning, as palliative care does not seem appropriate in these populations [17].

Murtagh et al. refined this method in 2014 because, according to the authors, the minimal method Rosenwax et al. described underestimates the number of people who may need palliative care. Additional diagnosis codes were added and others were eliminated to account for changes in diagnostic and treatment practices. Benign neoplasms were excluded, and chronic heart diseases, hypertensive and ischemic heart diseases, and cerebrovascular diseases were included. The categories of renal and liver failures were extended to include chronic renal and liver failures as well. Other chronic, respiratory, and neurodegenerative diseases were included, like multiple sclerosis and progressive supranuclear palsy. Additionally, all types of dementia and Alzheimer’s diseases were added to the Rosenwax classification [10].

The results of the Rosenwax method were contrasted with the Murtagh method, based on deaths in England occurring between 2006 and 2008. The comparison showed an increase from 37 to 63 % for all cases of death that were potentially in need of palliative care [10].

## Objectives

In Germany, no comprehensive population study exists on the demand for palliative care and its need within the population. Therefore, the aim of our study was to obtain a range of estimations to get a sense of how many people may be in need of palliative care. These data can be seen as baseline data for health system planning and are the basis for further discussion about palliative care needs in cancer and non-cancer patients. The current study was based on Murtagh et al. and Rosenwax et al.’s methods. The findings were then contrasted with international data.

## Method

This study was based on three (Rosenwax’s minimal and maximal estimations and Murtagh’s adapted version) of the existing methods to estimate the number of people who are possibly in need of palliative care. Data on the number and causes of deaths were derived from death registration data. This population-based database, published annually by the Federal Bureau of Statistics, is a complete inventory count of all cases of death in Germany. It contains information on the cause of death, as well as sex and age. The cause of death is coded using the ICD-10-WHO code, making international comparisons possible [18]. Based on the death certificate, qualified coders identify the underlying cause of death and document the relevant code. According to the WHO, the mortality statistics contain the main underlying cause of death (monocausal) [19]. The mortality statistics can be seen as secondary statistics and can be used for epidemiological research, regional comparisons, and health services research. As it is not possible in Germany to match the mortality statistics with hospital admission data, our analyses were restricted to the Rosenwax minimal and maximal estimate method [17] and the Murtagh method [10], an adaption of the Rosenwax minimal estimate method, performed by Murtagh et al. The database used for our calculation is the German Mortality Statistics 2013, as it is the latest data year available at this point.

To transfer the Rosenwax (Australia) and Murtagh (Great Britain) methods to German data, some adaptions had to be made to convert the defined codes to ICD-10-WHO year 2013 codes. This has been necessary, as the ICD-Codes between countries and years can differ (See Table 1 for the utilized ICD-10-WHO year 2013 codes). To apply the Rosenwax method to German data, the ICD-10-AM codes had to be converted to ICD-10-WHO codes. The ICD-codes our analyses are based on can be found in Table 1.

**Table 1:** Comparison of the Rosenwax and Murtagh methods for estimating the need for palliative care

Rosenwax Method		*n*	Murtagh Method		*n*	Difference
Neoplasm	C00–D48	230.840 (25.82 %)	Malignant neoplasm	C00–C97	223.842 (25.04 %)	6.998
			Cancer (breast)	C50	18.009 (2.01 %)	
			Cancer (colorectal)	C18–C21	25.693 (2.87 %)	
			Cancer (lung)	C30–C39	46.896 (5.25 %)	
			Cancer (prostate)	C61	13.408 (1.5 %)	
			Cancer (other)		119.836 (13.41 %)	
Heart failure	I110,I119, I500,I501,I509, I130,I132	74.704 (8.36 %)	Heart disease (chronic)	I00–I52	274.821 (30.75 %)	258.673
			Cerebrovascular disease (stroke)	I60–I69	58.556 (6.55 %)	
Renal failure	N10, N11, N18, N120, N131, N132	8.588 (0.96 %)	Renal disease (chronic renal failure)	N17, N18, N28, I12, I13	18.817 (2.11 %)	10.229
Liver failure	K704, K711, K721, K729	493 (0.06 %)	Liver disease	K70–K77	15.255 (1.71 %)	14.762
Chronic obstructive pulmonary disease	J40, J410, J411, J418, J42, 430–J449	31.570 (3.53 %)	Respiratory disease	J06–J18, J20–J22, J40–J47 & J96	55.557 (6.22 %)	23.987
Neurodegenerative disease	G10, G122, G20	10.841 (1.21 %)	Neurodegenerative disease	G10, G20, G35, G122, G903, G231	12.349 (1.38 %)	1.508
Huntington’s disease	G10	332 (0.04 %)				
Motor neuron disease	G122	1.878 (0.21 %)				
Parkinson’s disease	G20	8.631 (0.97 %)				
Alzheimer’s disease	G300, G301, G308, G309	6.252 (0.70 %)	Dementia, Alzheimer’s, senility	F01, F03, G30, R54	37.683 (4.22 %)	31.431
HIV/AIDS	B20–B24	401 (0.04 %)	HIV/AIDS	B20–B24	401 (0.045 %)	0
Total deaths from these conditions		363.689 **40.7 %** of all deaths (*n* = 893.825)	Total deaths from these conditions		697.281 **78.0 %** of all deaths (*n* = 893.825)	

A transformation of the Rosenwax ICD-10-AM to ICD-10-WHO codes was not possible for codes I111, N102, and N112. Hence, we replaced these codes with codes I11.0, I11.9, N10, N11.1, N11.8, and N11.9 ICD-10-WHO

The maximal estimate codes, defined by Rosenwax et al. and used by Murtagh et al., were converted into ICD-10-WHO codes that did not need any adaptions. The maximal estimate includes all deaths except those due to one of the following conditions:

- Pregnancy, childbirth, or puerperium (ICD-10-WHO codes O00–O99)
- Those originating during the perinatal period (ICD-10-WHO codes P00–P96)
- Injury, poisoning, and other similar causes (ICD-10-WHO codes S00–T98)
- External causes (ICD-10-WHO codes V01–Y98)

The database provided by the Federal Bureau of Statistics contains no data on the personal level, and is therefore open to the public. Thus, obtaining ethical approval is not necessary.

## Results

A total of 893,825 people died in Germany in 2013, with the majority dying from diseases of the circulatory system (39.7 %), followed by neoplasms (25.0 %). According to the Rosenwax method, a total of 363,689 (40.7 %) deaths were potentially in need of palliative care in Germany in 2013. Neoplasms accounted for 63.5 % of all deaths with regard to all potential palliative care candidates identified using the Rosenwax method.

Using the Murtagh method, the number of people who could benefit from palliative care was 697,281 (78.0 %), which is almost twice as many people compared to the Rosenwax method. The largest deviation between the Murtagh and Rosenwax methods could be found within the category “heart,” where almost 60.0 % of the deviation can be explained. The proportion of patients in need of palliative care because of a heart disease rises from 8.4 % (Rosenwax) to 30.8 % (Murtagh), making heart disease the most frequent underlying cause for the potential demand of palliative care.

**Fig. 1:**
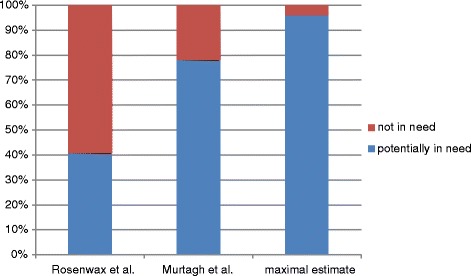
Percentage of death cases classified as potentially in need vs. not in need

According to the maximal estimate originally developed by Rosenwax et al. [17], a total of 858,546 people, accounting for 96.1 % of all deaths, could be potential palliative care patients. The Rosenwax minimal method identifies 40.7 % of all deaths eligible for palliative care, and Murtagh’s adaption of the Rosenwax method for a minimal estimate expands the demand to 78.0 % of all deaths (Fig. 1).

**Fig. 2:**
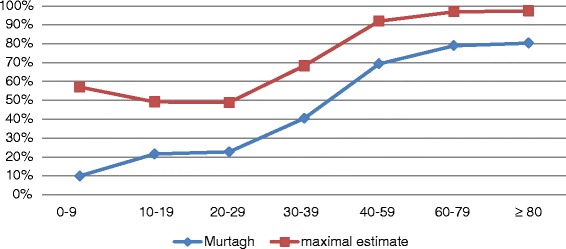
Demand for palliative care by age group

**Fig. 3:**
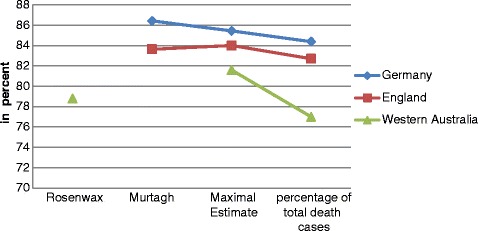
Demand for palliative care in death cases > 65 years of age incomparison to Rosenwax and Murtagh

To give further insight into age-related differences regarding the demand for palliative care, we performed an additional analysis accounting for the factor "age". We estimated the demand for palliative care within the different age groups with the Murtagh method and the maximal estimate (Rosenwax method). The results showed that, according to Murtagh, the demand in children aged 0 to 9 years lies at 9.9 % of all deaths occurring in this age category. The percentage of potential palliative care candidates rises to 40.4 % in the age category between 30 and 39 years and keeps increasing to 80.3 % in people aged 80 and over. Similar results, but on a higher level (10–15 percentage points higher than the Murtagh method), can be found for the maximal estimate, with the biggest discrepancies within the age category 0 to 9 years (almost 25 percentage points higher). The increase of potential palliative care needs with older age is caused by a change of the cause of death from more external causes of death, like accidents, to more cases of death caused by diseases, potentially creating a need for palliative care (e.g. cancer and vascular diseases) (Figs. 2 and 3).

## Discussion

Comparing the need for palliative care in the German population, we can find similar results to those from Western Australia and Great Britain. By using the Rosenwax method, we identified 40.7 % of all deaths occurring in Germany in 2013 as being caused by conditions Rosenwax et al. defined. Murtagh et al. found similar results for England, indicating that 37 % of all deaths may have been preceded by a need for palliative care [10]. The percentage, according Rosenwax et al.’s minimal estimate, is 50 % of all deaths for Western Australia [17]. Worldwide, the percentage ranges from between 25 % (Mexico) and 49 % (Netherlands), whereas in Western Europe (excluding Germany), the lowest rate lies at 41 % (Wales) [20]. Murtagh et al. adapted Rosenwax’s minimal estimate method by adding extra conditions that, in the opinion of questioned experts, palliative care seemed to be appropriate for. Other conditions where palliative care did not seem appropriate were deleted [10]. For England, an estimated 63 % of all deaths fall in the categories defined by Murtagh et al [10]. By using the Murtagh method, 78 % of all deaths in Germany would potentially be in need of palliative care. This percentage is substantially higher than the findings from England. Here, the highest discrepancy was found with regard to coded deaths due to heart disease. In Germany in 2013, the underlying cause of death was a heart disease in approximately 31 % of all cases, whereas in England, only about 12 % of all deaths were caused by heart disease [10]. Comparing our data to mortality data from the WHO in 2013, Germany has an age-standardized death rate for diseases of the circulatory system of 145.5 per 100,000, compared to the United Kingdom (104.3) or the United States (131.2) [21]. In addition to the higher mean age of the German population, differences in the population’s specific death rate for the circulatory system could partly explain the discrepancies found. Comparing the need for palliative care with data from Australia [17] and Great Britain [10] for people dying at an age over 65, Germany seems to have the highest demand for palliative care in this age group. In Germany, people dying over the age of 65 account for 84.4 % of all death cases, while in Western Australia only 77.0 % of deaths occur over the age of 65 [17].

With a median age of 46.5 years, Germany is the second oldest nation worldwide, with only Japan having a higher median age [22]. With older age, more and more people die from conditions potentially generating a need for palliative care; therefore, in an aging society, a higher demand for palliative care can be assumed.

The need for palliative care has to be seen alongside with the supply of palliative care in the corresponding country. Prior estimations for Germany stated a demand of 80 to 100 hospital beds (hospice and palliative care) per million inhabitants [13]. Inpatient palliative care is one of many ways to deliver palliative care. As the majority of Germans with terminal diseases wish to die at home [23], there is a need to provide palliative care at home. Because patients benefit from early access to palliative care [24, 25] and costs can be cut [26], it is important to implement palliative care early in care trajectories, making the demand for palliative care even higher, as the utilization is not restricted to the time shortly before death. Until now, it was mainly patients with cancer who received palliative care in Germany [27], although many terminally ill patients with a non-cancer disease benefit from palliative care as well [5–7].

## Limitations

The Rosenwax and the Murtagh methods are based on death certificate data. The limitations of the Rosenwax and Murtagh methods have been widely discussed [10, 17, 28]; therefore, at this point, we only want to give a short summary of the main limitations. Both measures are based on death certificate data, whereby some diagnoses (e.g. dementia, Parkinson’s or renal diseases) may be under-recorded [10], especially when only the underlying cause of death has been documented. Population-based measures, like the ones Rosenwax and Murtagh utilized are condition-based and are therefore not able to measure the patients’ actual needs for palliative care [10, 17, 28], as need is determined by many factors, in addition to the diagnosis alone [29, 30]. Severity, in addition to illness trajectories, is not accounted for. This may be especially relevant when it comes to severely ill children and adolescents, where there can be a possible demand for palliative care over the course of years [10]. These estimations on a population basis can be seen as a low-cost, pragmatic approach for policy and resource allocation, including a certain degree of misclassification [28].

If further data are available, other methods do exist to perform estimations on the realistic need for palliative care in a population. For example, Gómez-Batiste et al. performed an analysis based on the prevalence of symptoms, chronic conditions, and structural information regarding the rate of home residences [31]. Rosenwax et al. performed further estimations by linking hospital admission data to get a mid-range estimate, including all deaths with a hospitalization record one year prior to death because of the same condition that was documented in the death certificate [17]. As it is not possible to link further data to this nationwide German dataset, our results serve as a rough estimation of the palliative care needs in Germany. Further studies, based on health insurance companies’ data, could provide further information, although these results can lack generalizability. Our study is based on data of one single year (2013). Further findings can be obtained using a longitudinal analysis and expansion of the databases to more than one analyzed year.

## Conclusion

These figures (i.e. the amount of non-cancer patients who could benefit from palliative care) can provide clues to guide the future planning of palliative care service delivery for patients with non-cancer diseases – particularly since there is evidence of shortcomings in the provision of palliative care for non-cancer patients in Germany [7].

From 2009 to 2050, the number of deaths in Germany is expected to rise by 25 %, with a total increase of death cases and more people dying at an advanced age. With a higher need for palliative care at an older age, palliative care becomes more and more important [32].

After all, "the demand for palliative care" cannot be seen as a given fact, but is partly subject to continuously evolving ideas of what is appropriate care, economic resources and resource allocation. The estimates performed can serve as a rough approximation, highlighting the immense need for palliative care in an aging society like Germany.
